# Effects of green light emitting diode light during incubation and dietary organic macro and trace minerals during rearing on tibia characteristics of broiler chickens at slaughter age

**DOI:** 10.1016/j.psj.2020.11.042

**Published:** 2020-11-28

**Authors:** B.C. Güz, R. Molenaar, I.C. de Jong, B. Kemp, M. van Krimpen, H. van den Brand

**Affiliations:** ∗ Adaptation Physiology Group, Wageningen University and Research, 6700 AH, Wageningen, The Netherlands; † Wageningen Livestock Research, Wageningen University and Research, 6700 AH, Wageningen, Gelderland, The Netherlands

**Keywords:** green LED, incubation, organic mineral, tibia, broiler chicken

## Abstract

This study was designed to evaluate the effects of green light emitting diode (**LED**) light during incubation and dietary organic macro and trace minerals during rearing on tibia morphological, biophysical, and mechanical characteristics of broiler chickens at slaughter age. The experiment was setup as a 2 × 2 × 2 factorial arrangement, with the following treatments: 1) light during incubation (green LED light or darkness), 2) macro mineral source during rearing (organic or inorganic Ca and P), and 3) trace mineral source during rearing (organic or inorganic Fe, Cu, Mn, Zn, and Se). A total of 2,400 eggs (Ross 308) were either incubated under green LED light (16L:8D) or in complete darkness. After hatch, a total of 864 male broiler chickens were reared until slaughter age (day 42) and provided with 1 of 4 diets, differing in macro and/or trace mineral source. During rearing, the experiment had a complete randomized block design with 9 replicate pens per treatment and 12 chickens per pen. At slaughter age (day 42), 2 chickens per replicate were randomly selected and tibia bones were obtained. Tibia weight, length, thickness, osseous volume, pore volume, total volume, mineral content, mineral density, ultimate strength, and stiffness were determined. Green LED light during incubation did not affect any of the tibia characteristics. Dietary organic macro minerals positively affected most of the tibia morphological, biophysical, and mechanical characteristics compared to the inorganic macro minerals, whereas trace mineral sources did not affect tibia characteristics. It can be concluded that dietary organic macro minerals Ca and P stimulated tibia characteristics, whereas green LED light during incubation and dietary trace minerals during rearing did not affect tibia characteristics, locomotion, or leg disorders.

## Introduction

Increased attention is being focused on improving leg health of broiler chickens, due to an imbalance between high growth rate and immature bones and joints (Kestin et al., 1992, 2001; Knowles et al., 2008; Sherlock et al., 2010; Güz et al., 2019, 2020). Suboptimal leg health is known to result in pain and thereby negatively affects welfare and natural locomotion-related behaviors, such as accessing water and feed, especially in the last weeks of broilers rearing period (Mckay et al., 2000; Bradshaw et al., 2002; Bessei, 2006; Gocsik et al., 2017). In terms of economy, suboptimal leg health can cause higher mortality, lower slaughter revenues, and consequently results in financial losses (Kestin et al., 1999; Mench, 2004).

Two important factors, among others, that seem to affect bone development in broilers are light during incubation (Huth and Archer, 2015a, Huth and Archer, 2015b; Van der Pol et al., 2017, 2019) and macro and/or trace mineral sources during rearing (Hemme et al., 2005; Ferket et al., 2009; El-Husseiny et al., 2012; Güz et al., 2019).

In commercial hatcheries, eggs are mostly incubated under complete darkness (Archer et al., 2009; Tong et al., 2018). However, in nature, most developing avian embryos are regularly exposed to daylight for short periods of time when the hen leaves her nest to eat and drink (Archer, 2017; Archer and Mench, 2017) and when she rotates her eggs several times in a day (Duncan et al., 1978; Archer et al., 2009; Archer and Mench, 2014, 2017). It is known that light exposure during incubation affects chicken embryo development (Rozenboim et al., 2004; Özkan et al., 2012; Van der Pol et al., 2015, 2017), because broilers have a light-sensitive pineal gland, which produces melatonin (Aige-Gil and Murillo-Ferrol, 1992; Archer et al., 2009). Melatonin influences skeletal development in chickens (Archer et al., 2009; Huth and Archer, 2015a, Huth and Archer, 2015b; Archer and Mench, 2017).

In addition to light itself, the color of light might be an important factor, since chickens have special extraretinal photoreceptors in their eyes and brains (Lewis and Morris, 2000; Rozenboim et al., 2004; Sobolewska et al., 2019). Green is a common color in the natural habitat of avian species and eggs are exposed to green light when the nest is located beneath green leaves. Several studies evaluating green light emitting diode (**LED**) light during incubation in broiler chickens showed higher body weight and post-hatch pectoral muscle growth compared to complete darkness during incubation (Rozenboim et al., 2004; Halevy et al., 2006; Zhang et al., 2014, 2016), as well as higher liver weight, higher antioxidant activity, and higher melatonin levels (Zhang et al., 2014). Because of the effect of green light on melatonin levels, an effect of incubation with green light on bone development can be expected, but, to our knowledge, this has not been investigated yet.

Once embryonic bone development is stimulated by green light, it is necessary to provide chickens with sufficient minerals for further bone development during rearing as well. This means that bioavailability of minerals is important for optimal bone development during the rearing period (Bao and Choct, 2009; Yenice et al., 2015). Organic macro (Ca and P) and trace (Fe, Cu, Mn, Zn, and Se) minerals have been shown to have a higher bioavailability than inorganic minerals in broilers' diet, due to higher intestinal absorption of those minerals (Wedekind et al., 1991; Bao and Choct, 2009). This higher bioavailability resulted in higher bone mineralization in broiler chickens and better bone quality (Oviedo-Rondon et al., 2006; Ferket et al., 2009; El-Husseiny et al., 2012; Güz et al., 2019). Most studies related to organic minerals in broilers' diets have focused on trace minerals only (Ferket et al., 2009; Zhao et al., 2010; El-Husseiny et al., 2012), or on combinations with organic macro minerals (Ca and P) (Güz et al., 2019), but effects of organic macro minerals only are less clear.

The main objective of this study was to investigate the effect of green LED light during incubation and dietary organic macro and/or trace minerals during rearing on: 1) tibia characteristics, 2) locomotion, and 3) leg disorders at slaughter age in fast-growing broiler chickens.

## Materials and methods

### Experimental Design

The incubation phase of the experiment was conducted at Wageningen University & Research (Wageningen, The Netherlands). The rearing phase was conducted at the experimental facility of a commercial company (ForFarmers N.V., Lochem, The Netherlands). All procedures in this study were approved by the Central Commission for Animal Experiments (The Hague, The Netherlands; approval number AVD401002016686).

The experiment was setup as a 2 × 2 × 2 factorial arrangement with 2 phases (incubation and rearing). Factors included were 1) light during incubation (green LED or complete darkness), 2) macro mineral source (inorganic vs. organic), and 3) trace mineral source (inorganic vs. organic) during rearing. In total, there were 8 treatments and each treatment was replicated 9 times during the rearing phase. As a result, a total of 72 experimental pens within a complete randomized block design were used. Within each block of 8 pens, treatment groups were randomly distributed. Pen was used as the experimental unit and each pen contained 12 male broiler chickens.

### Incubation Phase

**Figure 1:**
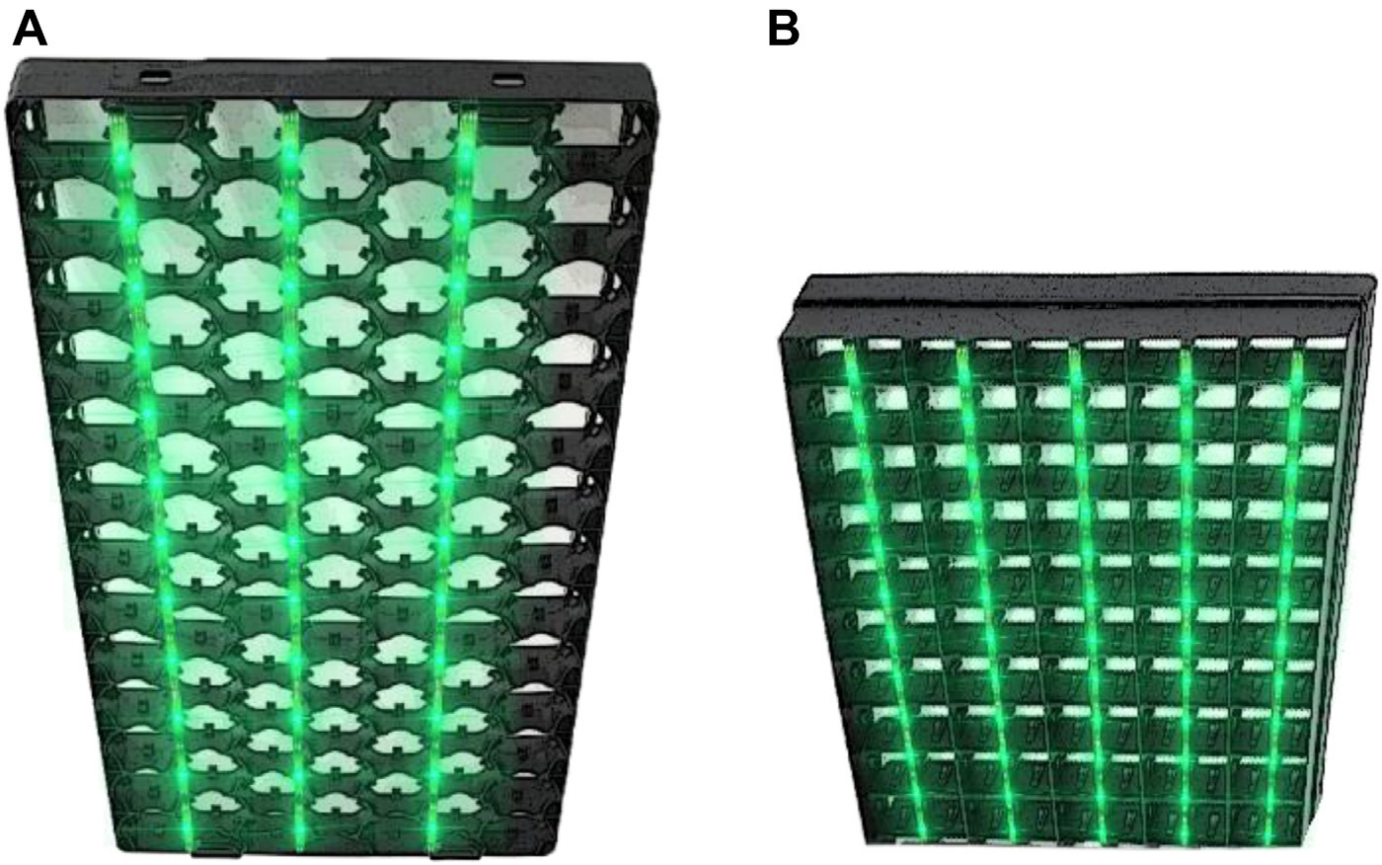
Assembling of green light emitting diode light strips beneath egg trays for the (A) large and (B) small incubator.

A total of 2,400 Ross 308 eggs from a 44-week-old breeder flock were obtained from a commercial hatchery (Lagerwey, Lunteren, The Netherlands). All eggs were selected based on weight between 61.0 and 65.0 g, transported from the hatchery to the research facility of Wageningen University & Research (Wageningen, The Netherlands), and stored at 18.0°C for 48 h. After storage, the eggs were randomly distributed over 4 incubators: 2 large incubators, containing 960 eggs each, and 2 small incubators, containing 240 eggs each. In the large incubators, trays contained 88 eggs in a honey structure. In the small incubator, trays contained 150 eggs in a row structure (see Figure 1). Per incubator, 4 or 5 eggshell temperature sensors (Pt-100, Sensor Data BV, Rijswijk, The Netherlands) were attached to 4 or 5 individual eggs. All sensors were placed at the equator of the chosen eggs by using heat conducting paste (Dow Corning 340 Heat Sink Compound, Dow Corning GmbH, Wiesbaden, Germany) and a small piece of tape (2 × 2 cm). The incubator temperature was continuously adjusted based on the median eggshell temperature to maintain an eggshell temperature of 37.8°C throughout incubation. Eggs were turned every 30 min at an angle of 90°. At embryonic day 8 (**E8**), all eggs were candled and infertile eggs were removed. At E18, all eggs were candled again and eggs containing a vital embryo were transferred from the trays into hatching baskets, which were placed back in the same incubator.

At E21, chicks were taken from the hatching baskets and feather-sexed. Chick quality parameters (red hock, red beak, and navel score) of all hatched chicks (both males and females; n = 1,810) were assessed. Red hock and red beak were scored as 0 (absent) or 1 (present). Navel score was assessed as 1 (good), 2 (moderate), or 3 (poor). Thereafter, 30 female chicks per treatment (darkness and green LED light) were randomly selected and body weight, residual yolk weight, yolk-free body mass, heart weight, liver weight, stomach weight, and intestinal weight were measured. A total of 864 male chicks were individually weighed and tagged with a neck label, vaccinated against infectious bronchitis (eye drop; MSD Animal Health, Boxmeer, The Netherlands), and transported for half an hour in a climate-controlled van to the rearing facility (Nijkerk, The Netherlands).

### Green LED Setup

Two incubators (1 large and 1 small) were equipped with plastic-covered and water-resistant green LED strips (Josef Barthelme Green LED strips, Josef Barthelme GmbH & Co., Nürnberg, Germany). These strips were attached underneath hatching trays, meaning that eggs underneath the hatching tray were lighted (Figure 1). Light intensities at eggshell level (approximately 7 cm distant from the LED strip), measured by a Lux meter (Testo Model 540 Lux meter, Testo BV, Almere, The Netherlands), were on average 288 lux. Green light was applied with a 16L:8D light schedule throughout incubation.

At E18, after transfer of eggs to the hatching baskets, LED strips were attached to metal frames holding the hatching baskets to maintain the green lighting during the last 3 d of incubation.

### Spectrum Measurement Through Eggshell

**Figure 2:**
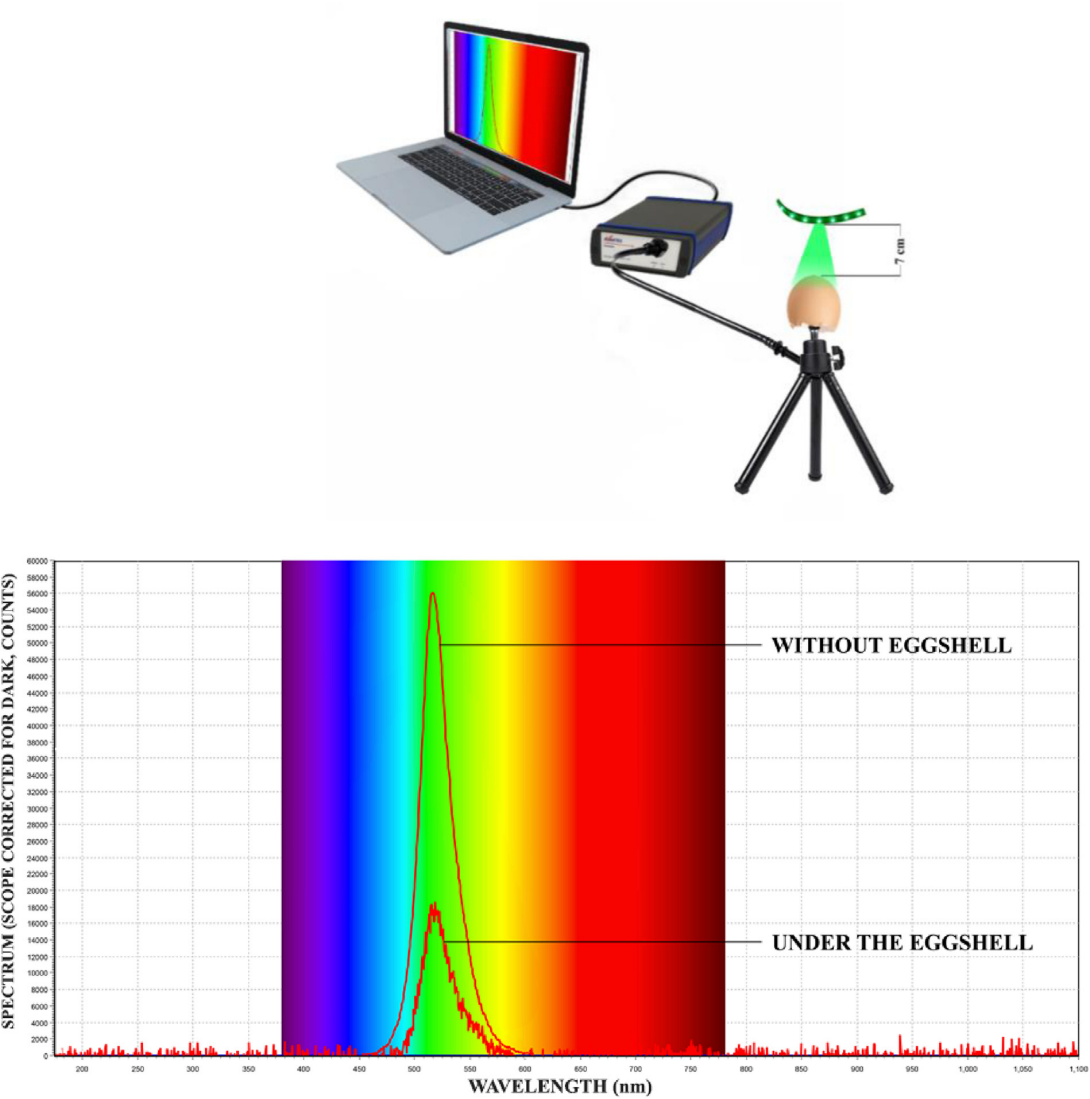
Unfiltered spectrum measurements, using the LED spectrometer, without eggshell (direct impose) and through the eggshell of broiler breeder eggs, illuminated by the green LED light strips used in the incubators. Abbreviation: LED, light emitting diode.

To determine the light spectrum of the green LED light passing through the eggshell, 16 Ross 308 broiler breeder eggs were chosen from the aforementioned collected eggs and egg contents were removed (shell membranes remained). The shells were placed individually over a LED spectrum meter sensor (AvaSpec ULS2048 Spectrometer, Avantes, Apeldoorn, The Netherlands). Green LED strips were placed 7 cm distant from the eggshell (comparable to the distance during incubation). The light spectrum passing through the eggshells and average spectrum were determined. The peak in light spectrum without an eggshell was obtained at a wavelength of 522 nm at approximately 57,000 counts, whereas the peak in light spectrum passing through the eggshell was obtained at a wavelength of 536 nm at approximately 19,000 counts (Figure 2).

### Rearing Phase

Upon arrival at the rearing facility, chickens were randomly assigned to 72 floor pens within 9 blocks of 8 pens in a climate-controlled broiler house. Each pen contained 12 male broiler chickens. Pen size was 0.90 m^2^ (1.2 × 0.75 m) and the floor was covered with 4 to 6 cm wood shavings. Temperature was maintained at 32°C until day 3 and thereafter gradually reduced to 22°C at day 42. A continuous light program from arrival to day 3 and a 16L:8D light program from day 4 to 42 were applied. Chickens were reared from arrival to day 42 with ad libitum access to feed and water. At day 11, chickens were individually vaccinated against Newcastle disease (Clone 30 eye drop, MSD Animal Health).

### Experimental Diets

A three-phase feeding program was applied; starter diets were provided from day 0 to 10, grower diets from day 11 to 28, and finisher diets from day 29 to 42. Dietary treatments were applied throughout all 3 phases. Four experimental diets were used in this experiment in a 2 × 2 factorial arrangement. They were: 1) inorganic macro and inorganic trace minerals; 2) inorganic macro and organic trace minerals; 3) organic macro and inorganic trace minerals; 4) organic macro and organic trace minerals. In the inorganic macro and organic trace minerals diet, inorganic sourced Ca and P were used, but the inorganic trace mineral premix was replaced by a 100% organic sourced trace mineral premix (Optimin, Trouw Nutrition, Tilburg, The Netherlands). In the organic macro and inorganic trace minerals diet, only the inorganic macro minerals (Ca and P), provided by limestone and monocalcium phosphate, were largely replaced by Calfos (Sonac Vuren B.V., Vuren, The Netherlands), an organic hydroxyapatite form of Ca and P source originating from processed porcine bones. An inorganic sourced trace mineral premix was used. In the organic macro and organic trace minerals diet, inorganic macro minerals were largely replaced by Calfos and the trace mineral premix with inorganic minerals was completely replaced by a complete organic sourced trace mineral premix (Table 1).

**Table 1:** Composition (%), and calculated and analyzed nutrients of the experimental diets (g/kg, as-fed basis).

Ingredients	Starter (0–10 d)				Grower (11–28 d)				Finisher (29–42 d)			
	IMIT	IMOT	OMIT	OMOT	IMIT	IMOT	OMIT	OMOT	IMIT	IMOT	OMIT	OMOT
Corn (%)	12.00	12.00	12.00	12.00	12.00	12.00	12.00	12.00	20.00	20.00	20.00	20.00
Soybean meal (%)	26.59	26.62	26.47	26.49	21.69	21.72	21.62	21.65	18.46	18.48	18.41	18.43
Wheat (%)	46.73	46.59	47.30	47.18	49.11	48.97	49.45	49.31	45.01	44.88	45.15	45.01
Rapeseed meal (%)	2.00	2.00	2.00	2.00	3.00	3.00	3.00	3.00	3.00	3.00	3.00	3.00
Soybean oil (%)	3.17	3.18	2.95	2.96	4.10	4.11	3.97	3.99	3.48	3.50	3.44	3.46
Sunflower seed meal (%)	3.00	3.00	3.00	3.00	4.00	4.00	4.00	4.00	5.00	5.00	5.00	5.00
Limestone (%)	0.72	0.83	-	0.08	0.59	0.70	0.15	0.27	0.30	0.41	0.15	0.27
Monocalcium phosphate (%)	0.84	0.83	-	-	0.48	0.47	-	-	0.15	0.14	-	-
Sodium chloride (%)	0.06	0.06	0.06	0.07	0.05	0.05	0.05	0.05	0.05	0.05	0.05	0.05
L-Threonine (%)	0.07	0.07	0.07	0.07	0.09	0.09	0.09	0.09	0.08	0.08	0.08	0.08
L-Lysine HCL (%)	0.29	0.28	0.28	0.28	0.32	0.32	0.32	0.31	0.30	0.30	0.30	0.30
Valine (%)	-	-	-	-	0.04	0.04	0.03	0.03	0.02	0.02	0.01	0.01
Xylanase (%)	0.10	0.10	0.10	0.10	0.10	0.10	0.10	0.10	0.10	0.10	0.10	0.10
DL-Methionine (%)	0.25	0.25	0.25	0.25	0.22	0.22	0.22	0.22	0.18	0.18	0.18	0.18
Soy lecithin (%)	1.50	1.50	1.50	1.50	1.50	1.50	1.50	1.50	1.50	1.50	1.50	1.50
Sodium bicarbonate (%)	0.38	0.37	0.38	0.37	0.36	0.35	0.36	0.35	0.36	0.36	0.36	0.36
Axtra PHY 5000L (%)	0.01	0.01	0.01	0.01	0.01	0.01	0.01	0.01	0.01	0.01	0.01	0.01
Oat hulls (%)	1.00	1.00	1.00	1.00	1.00	1.00	1.00	1.00	1.00	1.00	1.00	1.00
Inorganic minerals	1.00	-	1.00	-	1.00	-	1.00	-	1.00	-	1.00	-
Organic minerals	-	1.00	-	1.00	-	1.00	-	1.00	-	1.00	-	1.00
Maxiban	0.30	0.30	0.30	0.30	-	-	-	-	-	-	-	-
Salinomix	-	-	-	-	0.35	0.35	0.35	0.35	-	-	-	-
Calfos (%)	-	-	1.33	1.34	-	-	0.79	0.77	-	-	0.26	0.25
Total (%)	100.00	100.00	100.00	100.00	100.00	100.00	100.00	100.00	100.00	100.00	100.00	100.00
Calculated nutrients												
AMEn broiler (kcal/kg)	2,900	2,900	2,900	2,900	2,985	2,985	2,985	2,985	3,010	3,010	3,010	3,010
Crude protein (g/kg)	218	218	219	219	204	205	205	206	192	193	192	193
Crude fat (g/kg)	70	71	69	69	80	80	79	79	75	75	75	75
Crude fiber (g/kg)	35	35	35	35	37	37	37	37	37	37	37	37
Crude ash (g/kg)	59	59	56	55	51	51	49	49	44	44	44	44
Starch Brunt (g/kg)	356	355	359	359	370	369	372	371	396	396	397	396
Calcium (g/kg)	9.7	9.7	9.7	9.7	7.6	7.6	7.6	7.6	5.9	5.9	5.9	5.9
Phosphorus (g/kg)	6.0	6.0	5.9	5.9	5.2	5.2	5.2	5.2	4.4	4.4	4.4	4.4
Sodium (g/kg)	1.4	1.4	1.4	1.4	1.3	1.3	1.3	1.3	1.3	1.3	1.3	1.3
Copper (mg/kg)	19.3	19.3	19.1	19.1	19	19	18.9	18.9	18.7	18.7	18.7	18.7
Manganese (mg/kg)	120.8	121.1	117.3	117.3	120.5	120.9	117.9	118.8	116.7	117	115.4	116.4
Zinc (mg/kg)	89.2	89.2	89.1	89.1	89	89	88.9	88.9	88.7	88.7	88.6	88.6
6-Phytase E4a24 (FTU)	500	500	500	500	500	500	500	500	400	400	400	400
Dig lysine (g/kg)	11.5	11.5	11.5	11.5	10.8	10.8	10.8	10.8	9.9	9.9	9.9	9.9
Dig methionine (g/kg)	5.3	5.3	5.3	5.3	5.0	4.9	4.9	4.9	4.4	4.4	4.4	4.4
Dig methionine + cysteine (g/kg)	8.4	8.4	8.4	8.4	7.9	7.9	7.9	7.9	7.2	7.2	7.2	7.2
Dig threonine (g/kg)	7.1	7.1	7.1	7.1	6.8	6.8	6.8	6.8	6.3	6.3	6.3	6.3
Dig tryptophan (g/kg)	2.39	2.4	2.4	2.41	2.22	2.22	2.22	2.23	2.04	2.05	2.04	2.05
Dig isoleucine (g/kg)	7.7	7.7	7.7	7.7	7.0	7.0	7.0	7.0	6.5	6.5	6.5	6.5
Dig arginine (g/kg)	12.5	12.5	12.5	12.5	11.5	11.5	11.6	11.6	10.8	10.8	10.8	10.8
Dig valine (g/kg)	8.4	8.4	8.5	8.5	8.2	8.2	8.2	8.2	7.5	7.5	7.5	7.5
Dig glycine + serine (g/kg)	16.3	16.3	16.3	16.3	15.2	15.2	15.2	15.2	14.3	14.3	14.3	14.3
Retainable P broiler (g/kg)	4.4	4.4	4.4	4.4	3.7	3.7	3.7	3.7	2.8	2.8	2.8	2.8
Narasin (mg/kg)	50	50	50	50	-	-	-	-	-	-	-	-
Nicarbazin (mg/kg)	50	50	50	50	-	-	-	-	-	-	-	-
Salinomycin (mg/kg)	-	-	-	-	70	70	70	70	-	-	-	-
Analyzed nutrients												
Crude ash (g/kg)	56.4	55.4	52.7	53.9	56.3	51.8	50.1	49.5	41.8	41.1	42.1	42.3
Crude fiber (g/kg)	37.9	34.2	34.2	33.3	35.4	40.6	42.1	36.0	35.4	37.6	40.1	37.7
Crude protein (g/kg)	214.8	211.1	218.5	219.3	200.8	202.7	201.6	200.5	190.5	189.7	191.4	190.3
Starch (Brunt) (g/kg)	341.9	334.6	353.7	348.4	364.0	358.4	363.1	371.1	397.7	396.6	387.0	389.7
Crude fat (g/kg)	74.0	85.0	71.1	69.8	80.4	80.4	80.3	75.1	76.3	76.2	73.3	72.0
Calcium (g/kg)	9.8	9.6	9.8	9.7	7.7	7.8	7.7	7.6	6.0	6.1	6.0	6.1
Phosphorous (g/kg)	6.3	6.2	6.1	6.1	5.1	5.2	5.2	5.2	4.5	4.3	4.5	4.4
Sodium (g/kg)	1.2	1.3	1.3	1.4	1.2	1.2	1.2	1.2	1.2	1.1	1.2	1.2
Copper (mg/kg)	16.5	18.3	17.1	17.4	17.0	18.5	19.0	17.8	16.2	16.7	15.1	16.8
Iron (mg/kg)	35.4	35.2	34.8	36.1	37.6	35.9	35.8	35.4	34.1	36.3	34.7	35.6
Manganese (mg/kg)	100.8	113.9	108.6	109.6	122.5	116.0	119.2	114.1	104.3	113.9	112.8	112.2
Selenium (mg/kg)	32.5	30.8	30.9	31.5	33.5	32.7	31.5	32.4	31.5	31.8	30.9	33.1
Zinc (mg/kg)	91.2	94.1	89.7	90.8	87.1	93.2	90.1	91.9	87.1	88.1	84.2	87.3

Abbreviations: Dig, digestible; Ret, retainable; IMIT, inorganic macro plus inorganic trace minerals; IMOT, inorganic macro plus organic trace minerals; OMIT, organic macro plus inorganic trace minerals; OMOT, organic macro plus organic trace minerals.

^1^Phytase provided 1.22 g retainable phosphorus per kg at 500 FTU inclusion level (Guz et al., 2019).

^2^Composition of inorganic premix provided per kg of diet: 12,000 IU vitamin A (source of vitamin A), 2,400 IU vitamin D3, 30 IU vitamin E (source of vitamin E), 1.5 mg vitamin K3, 2 mg vitamin B1, 7.5 mg vitamin B2, 10 mg D-pantothenic acid, 35 mg niacin amide, 200 μg biotin, 20 μg vitamin B12, 1 mg folic acid, 3.5 mg vitamin B6, 461 mg choline chloride, 80 mg Fe (as FeSO_4_•H_2_O), 12 mg Cu (as CuSO_4_•5H_2_O), 60 mg Zn (as ZnSO_4_•H_2_O), 85 mg Mn (as MnO), 0.4 mg Co (as CoSO_4_•7H_2_O), 0.8 mg I (as KI), 0.1 mg Se (as Na_2_SeO_3_•5H_2_O), and 50 mg antioxidant (Guz et al., 2019).

^3^Composition of organic premix provided per kg of diet: 12,000 IU vitamin A (source of vitamin A), 2,400 IU vitamin D3, 30 IU vitamin E (source of vitamin E), 1.5 mg vitamin K3, 2 mg vitamin B1, 7.5 mg vitamin B2, 10 mg D-pantothenic acid, 35 mg niacin amide, 200 μg biotin, 20 μg vitamin B12, 1 mg folic acid, 3.5 mg vitamin B6, 461 mg choline chloride, 80 mg Fe (as Fe proteinate), 12 mg Cu (as Cu proteinate), 60 mg Zn (as Zn proteinate), 85 mg Mn (as Mn proteinate), 0.4 mg Co (as CoSO_4_•7H_2_O), 0.8 mg I (as KI), 0.1 mg Se (as Se selenite), and 50 mg antioxidant (Guz et al., 2019).

^4^Composition of Calfos provided per kg of product: 100 g crude protein, 300 g calcium, 130 g phosphorus (113 g retainable phosphorus), 50 g moisture (Guz et al., 2019).

All diets were produced and pelleted by ForFarmers N.V. and analyzed for ash (ISO 5984), dry matter (ISO 6496), crude fiber (ISO 6865), crude fat (ISO 6492), crude protein (ISO 5983), starch (ISO 6493), Na (ISO 6869), P (ISO 6941), Ca (ISO 6869), and Fe, Cu, Mn, Zn, and Se. Diet compositions and calculated and analyzed nutrient values are shown in Table 1.

### Data Collection, Sampling, and Measurements

All chickens were individually weighed on day 0, 10, 21, 27, 34, and 41. Feed intake (**FI**) was measured per pen for the starter, grower, and finisher period. Feed conversion ratios (**FCR**) were calculated for the 3 phases and over the whole growth period, taking mortality into account. Corrected FCR was calculated for the whole growth period, using the following equation:

$$CFCR=\text{FCR}+\frac{3,000-\text{ BW}}{3,333}$$

where CFCR = corrected FCR, FCR = feed conversion ratio, BW = body weight of all chickens per treatment, 3,000 = roughly the average of day 42 body weight (in g), and 3,333 = the correction constant (the correction for every 100 g of body weight is 0.03 points of FCR).

Mortality was recorded per pen per day. Gait score of 2 randomly selected chickens per pen was evaluated on day 27, 34, and 39 by using the method of Kestin et al. (1992) and scored within a range of 0 (normal locomotion) to 5 (unable to walk). To determine whether the light and diet factors might affect home pen behavior, observations were performed in morning and afternoon sessions on day 6, 13, 20, 27, 34, and 41, using the scan sampling technique (De Jong and Gunnink, 2019). During 3 to 4 min per session per day per pen, the number of chickens performing the following activities was scored: eating, drinking, walking, standing, resting, foraging, comfort behavior, dust bathing, and perching.

On day 42, 2 chickens per pen were randomly selected. Food pad dermatitis (**FPD**) was scored on both legs as 0 (no lesions), 1 (mild lesion), or 2 (severe lesion) (Ekstrand et al., 1998). Thereafter, chickens were subjected to electrical stunning, after which they were cut and bled. Breast weight, heart weight, and carcass weight were determined. Breast weight is described as the breast meat that was completely detached from the sternum. Carcass weight is defined as the weight of the body of the chicken without breast, legs, and wings. The left leg of each chicken was assessed by a veterinarian for bacterial chondronecrosis with osteomyelitis (**BCO**), epiphyseal plate abnormalities (**EPA**), and epiphysiolysis (**EPI**). All these leg abnormalities were scored in the range of 0 (no abnormalities), 1 (minor abnormality), or 2 (severe abnormality).

**Figure 3:**
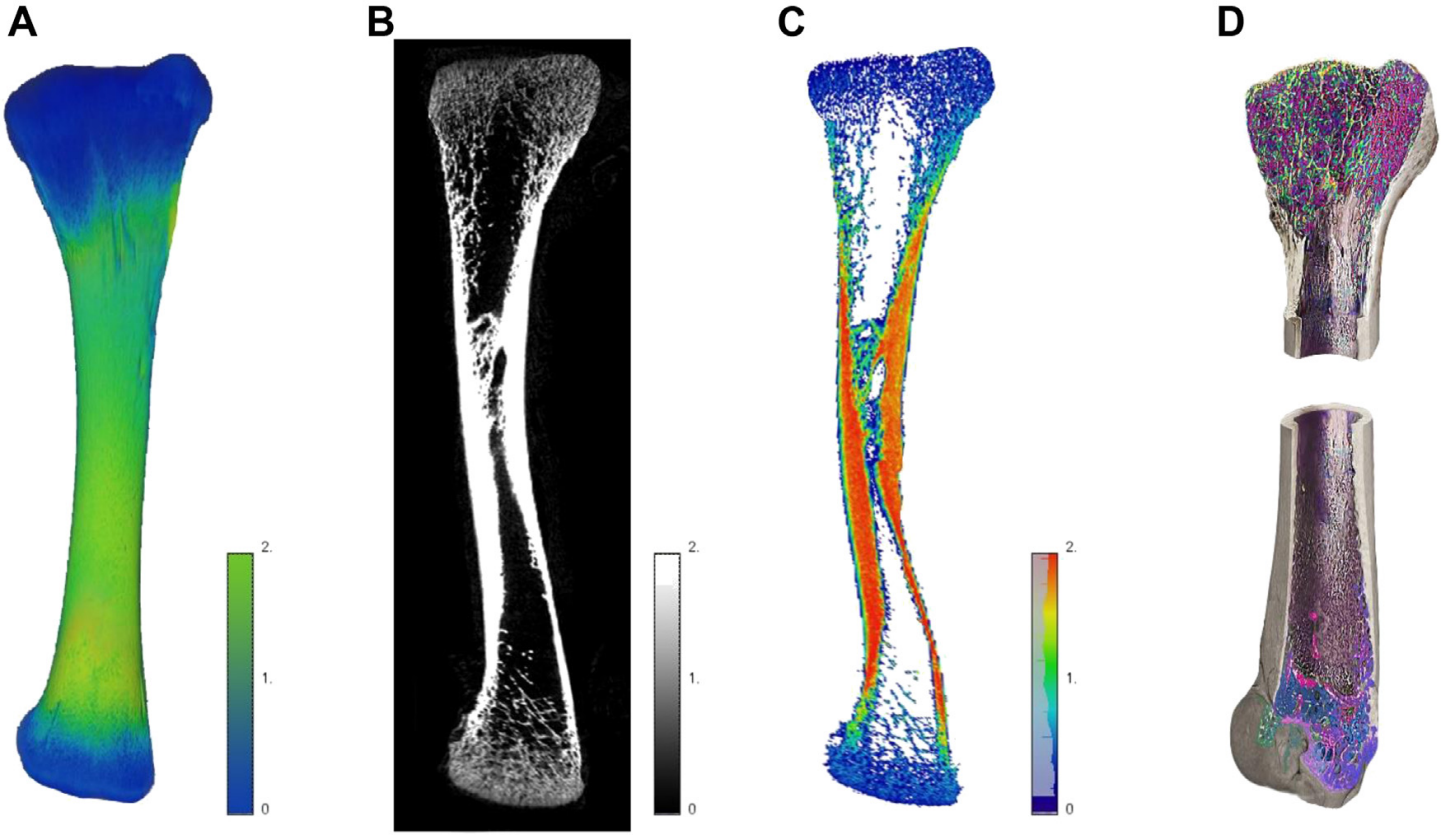
Illustration of scanned bone by 3D Micro-CT X-Ray and visualized in Avizo 3D viewer software (Thermo Fisher Scientific Inc., Waltham, Massachusetts). (A) Three-dimensional tibia outer view. Color scale represents the mineralization areas of bone from blue (less mineralization, 0) to green (more mineralization, 2). (B) Two-dimensional black and white (gray scale) tibia outer layer view. Shades of gray represent the mineralization areas of bone from dark gray (less mineralization) to white (more mineralization). (C) Two-dimensional colored tibia outer layer view. Color scale represents the mineralization areas of bone from blue (less mineralization, 0) to red (more mineralization, 2). (D) Three-dimensional tibia inner view scanned by 3D Micro-CT X-Ray scanner. Different colors represent different densities of bone materials and pores.

Right legs were deboned and tibias were obtained, packed, and frozen at −20°C. After thawing, tibia weight was determined. Tibia proximal length, lateral cortex thickness, femoral and metatarsal side proximal head thickness, osseous volume, pore volume, total volume (osseous volume + pore volume), volume fraction (osseous volume/total volume), mineral content, and mineral density were analyzed on individual tibia, using a GE Phoenix 3D X-ray microfocus CT scanner (General Electric Company, Boston, MA) (method described by Bouxsein et al., 2010) (Figure 3). Robusticity index was calculated, using the formula of Riesenfeld (1972): robusticity index (cm/g) = bone proximal length (cm)/bone weight (g).

The same tibias, scanned in three-dimensional X-ray measures, were subjected to a three-point bending test (method described by Jungmann et al., 2007), using an Instron electromechanical universal testing machine (Instron, Norwood, MA). Ultimate stress (maximal load of breaking point) data were used as the tibia ultimate strength; yield point (reached yield load just before the angle changed on slope, inflection point) data were used as the tibia yield strength; the slope of the selected linear part of the curve data was used as the tibia stiffness; the area under the curve of the selected region data was used as the tibia energy to fracture. Elastic modulus (GPa), which is the amount of strain as a result of a particular amount of stress and directly related to the density of bone (Novitskaya et al., 2011), was calculated using the following formula of Turner and Burr (1993):

$$E=\frac{N\;S^{3}}{4\delta \;TL^{3}}$$

where *E* is the elastic modulus (GPa), *N* is the maximal load (N), S is the span between bending fixtures (mm), T is the tibia thickness (mm), L is the tibia length (mm), and δ is the maximum deflection (mm) at the midpoint of the bone. For more details about X-ray and Instron-related bone variables, see Güz et al., 2019, Güz et al., 2020.

### Statistical Analysis

All statistical analyses were performed in SAS (version 9.4, 2013, SAS Institute Inc., Cary, NC).

Hatch data (red hock, red beak, navel score, residual yolk weight, yolk-free body mass, heart weight, liver weight, stomach weight, and intestines weight) were subjected to mixed model analysis, using the PROC MIXED procedure. Hatchling was used as the experimental unit and incubator was added to the model as a random effect.

The statistical model used was:

[1] Y=μ+Lightduringincubation+ε,

where Y = dependent variable, μ = overall mean, light = green LED light or complete darkness during incubation, and Ɛ = residual error.

All growth performance data (BW, FI, FCR, mortality), tibia morphological, biophysical, and mechanical characteristics, slaughter characteristics, and home pen behavior were subjected to mixed model analysis, using the PROC MIXED procedure.

The statistical model used was:

[2] Y=μ+Lightduringincubation+Macrominerals+Traceminerals+Interactions+ε,

where Y = dependent variable, μ = overall mean, light during incubation = green LED light or complete darkness, macro minerals = inorganic or organic Ca and P during rearing, trace minerals = inorganic or organic trace minerals during rearing, interactions = two-way and three-way interactions between light during incubation, macro minerals, and trace minerals, and Ɛ = residual error.

Pen was used as the experimental unit for all analyses (n = 72). The different blocks within the broiler house (n = 8) were used as a random effect. Body weight at slaughter age was added to the model as a covariable for tibia characteristics. Model assumptions were approved at both means and residuals. Non-normal distributed data were log-transformed before analyses. Results are provided as LSmeans ± SEM. When multiple comparisons were performed, the level of significance was corrected, using Bonferroni correction.

Gait score and leg disorders (FPD, EPA, BCO, and EPI) were subjected to generalized linear mixed model analysis, using the PROC GLIMMIX procedure, using model 2. Gait score, FPD, EPA, BCO, and EPI were analyzed at the multinomial level. Body weight at slaughter age was added to the model as a covariable for gait score and leg disorders. Effects were considered to be significant at *P* ≤ 0.05.

## Results

Only main effects are shown in all the tables (table 2, 3, 4) because only 1 three-way interaction and a few two-way interactions were found and these will be discussed in the text.

### General Hatch Data

Hatchability of fertile eggs was on average 87.8% and was not affected by green LED light or darkness during incubation (*P* = 0.11). Chick quality characteristics (red hock, red beak, and navel score of all chickens; and residual yolk weight, yolk-free body mass, heart weight, liver weight, stomach weight, and intestines weight of 30 female chickens per treatment) are shown in Supplementary Table 1 in the Supplementary data. No differences were found in any of these parameters between green LED light-incubated chickens and dark-incubated chickens (*P* > 0.06).

### Growth Performance

No two-way or three-way interactions between incubation light, macro mineral, and trace mineral source were found for performance characteristics from day 0 to day 42. Mortality was not affected by any of the treatments (Table 2).

**Table 2:** Effects of green LED light during incubation and dietary macro and trace mineral sources during rearing on body weight, feed intake, FCR (corrected for mortality), and mortality at different ages of male broiler chickens.

Parameter	Incubation		Macro minerals		Trace minerals		SEM	*P*-values		
	Darkness	Green	Inorganic	Organic	Inorganic	Organic		Incubation	Macro minerals	Trace minerals
Body weight day 0	42.7	42.6	42.6	42.6	42.6	42.6	0.11	0.43	0.98	0.97
Body weight day 10	305^b^	316^a^	307^b^	313^a^	312	309	2	<0.001	0.02	0.20
Body weight day 21	949	958	945	962	938^b^	969^a^	10	0.56	0.24	0.03
Body weight day 28	1,476	1,515	1,471^b^	1,520^a^	1,469^b^	1,523^a^	18	0.13	0.05	0.03
Body weight day 35	2,243	2,294	2,236^b^	2,302^a^	2,248	2,290	18	0.06	0.02	0.11
Body weight day 42	2,901^b^	2,994^a^	2,910^b^	2,984^a^	2,918	2,977	23	0.005	0.03	0.08
Feed intake day 0–10	280	278	281	278	281	277	3	0.48	0.33	0.29
Feed intake day 11–28	1,764^a^	1,738^b^	1,761	1,741	1,754	1,748	8	0.04	0.10	0.62
Feed intake day 29–42	2,504	2,523	2,525	2,502	2,544^b^	2,483^a^	26	0.24	0.18	<0.001
FCR day 0–10	1.07^b^	1.02^a^	1.06^b^	1.03^a^	1.05	1.05	0.02	0.004	0.03	0.88
FCR day 11–28	1.50	1.47	1.50	1.47	1.50	1.47	0.02	0.18	0.25	0.19
FCR day 29–42	1.72	1.67	1.71	1.68	1.73^a^	1.66^b^	0.03	0.09	0.33	0.02
FCR day 0–42	1.57^a^	1.51^b^	1.56^a^	1.51^b^	1.57^a^	1.51^b^	0.01	0.004	0.008	0.002
Corrected FCR day 0–42^1^	1.60^a^	1.52^b^	1.60^a^	1.53^b^	1.60^a^	1.53^b^	0.02	0.004	0.02	0.006
Mortality	4.6	5.6	4.6	5.6	5.8	4.6	0.02	0.42	0.43	0.31

^a,b^ Values within a row and factor lacking a common superscript differ (*P* ≤ 0.05).

Abbreviations: BW, average body weight of all chickens per treatment; CFCR, corrected FCR; FCR, feed conversion ratio; LED, light emitting diode.

1 CFCR was calculated as: CFCR = FCR + (3,000 − BW)/3,333.

At day 10 (Δ = 11 g; *P* < 0.001), day 35 (Δ = 51 g; *P* = 0.06), and day 42 (Δ = 93 g; *P* = 0.005) of age, chickens incubated under green LED light had or tended to have a higher body weight compared to chickens incubated under complete darkness (Table 2). FI between day 11 and 28 was lower in the green light-incubated chickens than in the complete darkness-incubated chickens (Δ = 26 g; *P* = 0.001). FCR between day 0 and 10 was lower in the darkness-incubated chickens (Δ = 0.05; *P* = 0.004) than in green light-incubated chickens. FCR (Δ = 0.06; *P* = 0.004) and corrected FCR (Δ = 0.08; *P* = 0.004) between day 0 and 42 were lower in green light-incubated chickens than in darkness-incubated chickens (Table 2).

At day 10 (Δ = 6 g; *P* = 0.02), day 28 (Δ = 49 g; *P* = 0.05), day 35 (Δ = 66 g; *P* = 0.02), and day 42 (Δ = 74 g; *P* = 0.03), chickens fed with organic macro minerals had a higher BW compared to chickens fed with inorganic macro minerals (Table 2). FCR between day 0 and 10 was lower in the chickens fed with inorganic macro minerals (Δ = 0.03; *P* = 0.03) than chickens fed with inorganic macro minerals. FCR between day 0 and 42 (Δ = 0.05; *P* = 0.008) and corrected FCR (Δ = 0.07; *P* = 0.02) were lower in chickens fed with organic macro minerals than in chickens fed with inorganic macro minerals (Table 2).

At day 21 (Δ = 31 g; *P* = 0.03) and day 28 (Δ = 54 g; *P* = 0.03), chickens fed with organic trace minerals had a higher BW compared to chickens fed with inorganic trace minerals (Table 2). FI between day 29 and 42 was higher in the chickens fed with inorganic trace minerals (Δ = 61 g; *P* < 0.001) than in the chickens fed with organic trace minerals. FCR between day 0 and 10 was lower in the chickens fed with organic macro minerals (Δ = 0.03; *P* = 0.03) than chickens fed with inorganic macro minerals.

### Tibia Morphological Characteristics

A three-way interaction between light during incubation, macro mineral source, and trace mineral source was found on tibia length. However, after applying Bonferroni correction, this interaction disappeared. No two-way interaction effects among light during incubation, macro mineral source, and trace mineral source were found for tibia morphological characteristics. No main effects of green LED light were found on tibia morphological characteristics. Main effects of macro and trace mineral source were found on tibia proximal length. Chickens fed with organic macro minerals (Δ = 0.27 cm; *P* = 0.04) or organic trace minerals (Δ = 0.21 cm; *P* = 0.01) had a higher tibia length than chickens fed with the inorganic varieties (Table 3).

**Table 3:** Effects of green LED light during incubation and dietary macro and trace mineral sources during rearing on tibia morphological, biophysical, and mechanical characteristics at day 42 of age in male broiler chickens (n = 2 chickens per pen; 9 pens per treatment; LSmeans).

Parameter	Incubation		Macro minerals		Trace minerals		SEM	*P*-values		
	Darkness	Green	Inorganic	Organic	Inorganic	Organic		Incubation	Macro minerals	Trace minerals
Tibia weight (g)	16.37	16.46	16.34	16.49	16.35	16.48	0.08	0.47	0.18	0.24
Tibia proximal length (cm)	12.70	12.55	12.49^b^	12.76^a^	12.52^b^	12.73^a^	0.07	0.15	0.04	0.01
Tibia lateral cortex thickness (cm)	1.33	1.31	1.30	1.34	1.33	1.32	0.01	0.21	0.07	0.43
Tibia head thickness femoral side (cm)	3.23	3.23	3.22	3.24	3.23	3.22	0.01	0.71	0.13	0.45
Tibia head thickness metatarsal side (cm)	3.04	3.03	3.03	3.04	3.04	3.03	0.01	0.86	0.47	0.36
Tibia robusticity index (length/weight)	0.78	0.76	0.77	0.77	0.76	0.78	0.01	0.11	0.48	0.19
Tibia osseous volume (cm^3^)	25.4	25.3	24.5^b^	26.1^a^	25.7	24.9	0.5	0.84	0.02	0.23
Tibia pore volume (cm^3^)	5.0	4.9	4.8	5.1	5.1	4.8	0.2	0.45	0.15	0.13
Tibia total volume (cm^3^)	30.4	30.1	29.3^b^	31.3^a^	30.9	29.7	0.5	0.69	0.02	0.13
Tibia volume fraction (OV/TV, %)	83	84	84	84	83	84	0.4	0.55	0.99	0.34
Tibia mineral content (g)	15.3	15.2	14.1^b^	16.3^a^	15.3	15.1	0.1	0.57	<0.001	0.25
Tibia mineral density (g/cm^2^)	0.32	0.32	0.27^b^	0.37^a^	0.32	0.32	0.01	0.86	<0.001	0.26
Tibia ultimate strength (N)	267	267	261^b^	272^a^	266	268	3	0.97	0.006	0.63
Tibia yield strength (N)	234	233	229^b^	237^a^	234	233	3	0.77	0.04	0.67
Tibia stiffness (N/mm)	228	224	220^b^	232^a^	227	225	2	0.34	0.002	0.73
Tibia energy to fracture (N-mm)	244	244	239^b^	249^a^	244	245	3	0.85	0.002	0.80
Tibia elastic modulus (GPa)	12.2	12.7	12.4	12.6	12.6	12.3	0.3	0.29	0.67	0.48

^a,b^ Values within a row and factor lacking a common superscript differ (*P* ≤ 0.05).

Abbreviations: LED, light emitting diode; OV, osseous volume; TV, total volume.

### Tibia Biophysical Characteristics

No three-way or two-way interactions between light during incubation, macro mineral source, and trace mineral source were found on tibia biophysical characteristics. Additionally, no main effects of green LED light and dietary trace mineral source were found on tibia biophysical characteristics. Chickens fed with organic macro minerals had a higher osseous volume (Δ = 1.6 cm^3^; *P* = 0.02), higher mineral content (Δ = 2.2 g; *P* < 0.001), and higher mineral density (Δ = 0.10 g/cm^2^; *P* < 0.001) compared to chickens fed with inorganic macro minerals (Table 3).

### Tibia Mechanical Characteristics

No two-way or three-way interactions between light during incubation, macro mineral source, and trace mineral source were found on tibia mechanical characteristics. No main effects of green LED light and dietary trace mineral source were found on tibia mechanical characteristics. Chickens fed with organic macro minerals had a higher ultimate strength (Δ = 11 N; *P* = 0.006), higher yield strength (Δ = 8 N; *P* = 0.04), higher stiffness (Δ = 12 N/mm; *P* = 0.002), and higher energy to fracture (Δ = 10 N-mm; *P* = 0.002) compared to chickens fed with inorganic macro minerals (Table 3).

### Locomotion-Related Observations (Leg Disorders and Gait Score), Slaughter Characteristics, and Home Pen Behavior

No two-way or three-way interactions between light during incubation, macro mineral source, and trace mineral source were found on gait scores and leg disorders. Chickens incubated under green LED light had higher gait scores on day 34 (Δ = 0.19; *P* = 0.02) and day 39 (Δ = 0.31; *P* = 0.001) compared to chickens incubated under complete darkness (Table 4). The incidence of leg disorders (FPD, BCO, EPA, and EPI) in all treatment groups was very low. EPA was not scored at all in any of the treatment groups. FPD, BCO, and EPI had an average score of 0.28, 0.10, and 0.20, respectively, and no significant differences were observed between treatment groups.

**Table 4:** Effects of green LED light during incubation and dietary macro and trace mineral sources during rearing on gait score at day 27, 34, and 39 of age in male broiler chickens (n = 2 chickens per pen; 9 pens per treatment; LSMeans).

Parameter	Incubation		Macro minerals		Trace minerals		SEM	*P*-values		
	Darkness	Green	Inorganic	Organic	Inorganic	Organic		Incubation	Macro minerals	Trace minerals
Gait score day 27^1^	2.27	2.27	2.28	2.28	2.25	2.27	0.05	0.85	0.92	0.81
Gait score day 34^1^	2.26^b^	2.45^a^	2.35	2.38	2.33	2.44	0.06	0.02	0.71	0.15
Gait score day 39^1^	2.70^b^	3.01^a^	2.90	2.81	2.85	2.82	0.07	0.001	0.36	0.88

^a,b^Values within a row and factor lacking a common superscript differ (*P* ≤ 0.05).

Abbreviation: LED, light emitting diode.

1 Method of Kestin et al. (1992), scored within a range of 0 (normal locomotion) to 5 (unable to stand).

Slaughter characteristics (breast weight, heart weight, and carcass weight) are presented in Supplementary Table 2 in the Supplementary data. Home pen behavior parameters (eating, drinking, walking, standing, resting, foraging, comfort behavior, dust bathing, and perching) for each of the scanning days (day 6, 13, 20, 27, 34, and 41) are presented in Supplementary Table 3 in the Supplementary data. No difference was found in any of slaughter characteristics and home pen behavior parameters among treatment groups.

## Discussion

### Green LED Light

Several environmental factors, such as temperature, humidity, ventilation, and egg turning, are known to play important roles on the development of chicken embryos (Decuypere et al., 2001; Molenaar et al., 2010). Light during incubation is another important factor related to embryonic muscular development (Aige-Gil and Murillo-Ferrol, 1992; Archer et al., 2009; Huth and Archer, 2015a, Huth and Archer, 2015b; Archer and Mench, 2017). Light can pass through the eggshell, as demonstrated in the current study, and as reported by Shafey (2004) and Shafey et al. (2005) with different wavelengths, meaning that embryos were indeed exposed to light during incubation. The current study showed higher BW in chickens at different ages incubated under green LED light compared to chickens incubated under darkness. This is in agreement with studies of Zhang et al., 2014, Zhang et al., 2016 and Rozenboim et al. (2004), wherein green LED light during incubation resulted in higher body weights and especially post-hatch pectoral muscle growth by improved proliferation and differentiation of satellite cells during embryonic development and post hatch period, compared to chickens incubated under complete darkness. Zhang et al. (2014) also reported higher liver weight, antioxidant activity, and melatonin levels in broiler chickens exposed to green LED light during incubation compared to darkness-incubated chickens.

Regarding bone development, green LED light during incubation has been found to increase bone development-related hormones, such as plasma growth hormone, prolactin, melatonin (Archer et al., 2009; Huth and Archer, 2015a, Huth and Archer, 2015b; Archer and Mench, 2017), hypothalamic growth hormone releasing hormone, growth hormone receptor, and insulin-like growth factor-1 of broiler chickens (Dishon et al., 2017). However, in the current study, no effect of green LED light during incubation was found on tibia morphological, biophysical, or mechanical characteristics at slaughter age. Results of this study suggest that although green light during incubation has been found to stimulate the above-mentioned hormones, receptors, and growth factors (Archer and Mench, 2017; Dishon et al., 2017), and to accelerate body weight gain (Rozenboim et al., 2004; Halevy et al., 2006; Zhang et al., 2014, 2016), its effect on bone development appears limited. It can be speculated that despite green LED light during incubation having a positive influence on growth factors and pectoral muscle growth, by enhancing the above-mentioned hormones and receptors, these influences do not seem sufficient to stimulate bone development.

Although no effect of green LED light during incubation was found on bone characteristics at slaughter age, gait score was worse in chickens incubated in green LED light compared to chickens incubated in darkness, even after correction for differences in BW. This suggests that factors other than bone characteristics (muscles, joints, tendons) may play a role as well in the locomotion of broiler chickens, but how these different leg parts interact with locomotion is still unclear. That other factors play a role in locomotion is also demonstrated by the almost complete lack of leg bone pathologies in the current study, whereas the gait score differed between treatments.

### Macro Minerals

This study showed that replacement of inorganic sourced macro minerals (Ca and P) by organic varieties resulted in higher body weight in most phases of the rearing period, while preserving similar levels of FI, which resulted in lower FCR. These findings are in line with previous studies, which reported that supplying organic macro minerals to broiler chickens stimulated growth performance (Bradbury et al., 2017, 2018). Organic sourced macro minerals have covalent bonds that provide better binding strength compared to electrovalent bonds in inorganic minerals. This enables these minerals to bind with other compounds more efficiently (Vieira, 2008; Bao and Choct, 2009), resulting in better chemical stability, and in greater resistance to pH changes throughout the digestive system (Selle at al., 2009; Wang et al., 2019). Finally, this results in lower antagonistic complex formation with minerals or other diet ingredients, resulting in higher bioavailability and an overall lower FCR.

Thus, replacement of inorganic macro minerals by a similar amount of organic macro minerals in the diet appears to result in comparable or even higher growth performance. Results of this study suggest that current broiler diets can be improved by increasing macro mineral availability for fast growing male broiler chickens to enhance growth performance. It can be questioned whether or not a further increase in inorganic macro minerals is desired, because of the potential risk of mineral complex formation and waste of minerals in the environment (Dozier III et al., 2003; Bao et al., 2007). Alternatively, the use of organic varieties of macro minerals might be a more sustainable solution.

The replacement of inorganic by organic macro minerals (Ca and P) did not only result in enhanced growth, but also in better bone characteristics. Almost all tibia morphological, biophysical, and mechanical characteristics were positively affected by the dietary organic macro minerals. Ca and P are known to be essential for bone development as a main component of the bone matrix (Rath et al., 1999; Blake and Fogelman, 2002), as an essential part of enzymes involved in bone development (McDevitt et al., 2006), and also as an essential part of bone development-related hormones, such as growth hormone, insulin-like growth factor-1, T3, and T4 (Parmer at al., 1987; Rosol and Capen, 1997). Increasing Ca and P levels in broiler chicken diets has been shown to positively influence bone mineralization, leading to stronger bones (Driver et al 2005a, Driver et al 2005b; Létourneau-Montminy et al., 2008). A deficiency of Ca or P may impair bone growth, mineral density, and strength (Bar et al., 2003; Sá et al., 2004; McDevitt et al., 2006; Dibner et al., 2007). Rapid increase in bone mineralization and bone growth during the rearing period of fast growing broilers, expressed in high tibia Ca and P levels, is crucial for leg bone strength (Shao et al., 2019). Onyango et al. (2003) reported that increasing dietary Ca and P resulted in increased tibia mineral content of broiler chickens. Guinotte et al. (1991) reported that organic sourced Ca in broiler chicken diets resulted in higher tibia proximal length and lateral cortex thickness compared to inorganic sourced Ca. This is in accordance with the results of the current study and overall it can be suggested that organic dietary Ca and P may provide better bone health to broiler chickens than inorganic Ca and P sources.

### Trace Minerals

The current study showed that replacement of inorganic trace minerals (Fe, Cu, Mn, Zn, and Se) by their organic counterparts resulted in (tendencies to) higher body weight during the rearing period. These findings are in in line with previous studies that showed that dietary organic sourced Cu, Fe, Mn, and Zn (Bao and Choct, 2009; Abdallah et al., 2009; Ao et al., 2009; Huang et al., 2009) resulted in higher growth performance than the inorganic varieties. This is probably because of increased bioavailability and less antagonistic complex formation with each other, as explained above. Yenice et al. (2015) showed that serum mineral concentrations of broiler chickens were higher by 22% for Mn, 17% for Zn, and 20% for Cu when minerals were provided in their organic form instead of the inorganic variety, which indicates that organic minerals have a higher bioavailability for chickens than inorganic minerals. This higher bioavailability is supported by findings that the difference in BW gain is particularly due to a higher feed efficiency, rather than due to an increase in FI. It appears that levels of inorganic trace minerals used in the current experiment might be suboptimal to reach the genetic potential in weight gain of fast growing male broiler chickens. However, a further increase of dietary trace minerals may result in more wastage of minerals in the environment and even toxic effects (Dozier III et al., 2003). Switching to organic trace minerals may reduce mineral losses in the environment (Nollet et al., 2007; Leeson and Caston, 2008).

Trace minerals are known to play major roles in metabolism, particularly in hormone and enzyme systems (Richards, 1997), among which some are related to embryonic and post-hatch bone development (Angel, 2007; Dibner et al., 2007). Cu has an essential role in bone strength and flexibility (Rucker et al., 1998; Dibner et al., 2007). Zn is essential for the functioning of bone osteoclast cells (Richards et al., 2010). Mn is required for the synthesis of bone cartilage (Eyre, 2004; Dibner et al., 2007). Fe, I, and Se are also essential trace minerals required for bone development by regulating thyroid functions, which are known to regulate bone growth (Jianhua et al., 2000; Medeiros et al., 2002; Röttger et al., 2011). Based on these functions in bone-related metabolism, it can be suggested that higher bioavailability of trace minerals, due to the use of organic trace mineral varieties, might improve bone characteristics. Although some previous studies, using organic trace minerals, have found a positive effect on bone development (Dibner et al., 2007; El-Husseiny et al., 2012), in the current study, tibia morphological, biophysical, and mechanical characteristics were not affected by trace mineral sources. These results are in agreement with other previous studies (Abdallah et al., 2009; Zhao et al., 2010). As a result of no effects of trace mineral sources on bone characteristics in this study, it can be speculated that the current guidelines for trace minerals are sufficient for bone development, whereas this does not appear to be the case for growth performance.

In conclusion, using green LED light during incubation, dietary organic macro minerals, and organic trace minerals resulted in higher BW gain of broiler chickens compared to darkness during incubation, inorganic macro minerals, and inorganic trace minerals, respectively. However, only dietary organic macro minerals positively affected most of the tibia morphological, biophysical, and mechanical characteristics. This suggests, on one hand, that macro mineral source rather than trace mineral source appears to be more important for leg bone development in current broiler chicken strains. However, on the other hand, effects of macro or trace mineral source on locomotion and leg pathologies appear to be marginal.
