# Supplementary material for: Effects of green light emitting diode light during incubation and dietary organic macro and trace minerals during rearing on tibia characteristics of broiler chickens at slaughter age
Source: Poult Sci. 2020 Nov 28;100(2):707–20. doi: 10.1016/j.psj.2020.11.042 (PMC7858192; doi:10.1016/j.psj.2020.11.042)
Supplement: Supplementary Appendix [file mmc1.docx]

**APPENDIX**

Table A1. Effects of green LED light during incubation on hatchling characteristics (red hock, red beak, navel score, residual yolk weight, yolk free body mass, heart weight, liver weight, stomach weight, and intestines weight) of female broiler chickens.

| Parameter^1^ | Incubation light condition | | SEM | P value |
| --- | --- | --- | --- | --- |
|  | Darkness | Green LED light |  |  |
| Chick weight (g) | 42.2 | 42.4 | 0.31 | 0.79 |
| Residual yolk weight (g) | 3.96 | 3.88 | 0.16 | 0.71 |
| Yolk-free body mass (g) | 38.3 | 38.5 | 0.28 | 0.60 |
| Heart weight (% of YFBM) | 0.81 | 0.84 | 0.02 | 0.18 |
| Liver weight (% of YFBM) | 2.94 | 2.98 | 0.05 | 0.60 |
| Intestines weight (% of YFBM) | 6.07 | 5.99 | 0.41 | 0.68 |
| Stomach weight (% of YFBM) | 6.00 | 6.28 | 0.14 | 0.06 |
| Red hock^2^ (%) | 1.8 | 2.4 | 0.01 | 0.36 |
| Red beak^2^ (%) | 6.3 | 2.9 | 0.02 | 0.07 |
| Navel score^2^ | 1.26 | 1.29 | 0.03 | 0.37 |

^1^ 30 chicks per treatment were assessed for all chick quality parameters, except for red hocks, red beaks, and navel score (n=864).

^2^ Red hock and red beak of all chicks were scored as 0 (absent) or 1 (present) and expressed as percentage chickens with score 1; navel score was assessed as 1 (good), 2 (moderate), or 3 (poor) and expressed as average score.

Table A2. Effects of green LED light during incubation and dietary macro and trace mineral source during rearing on average body weight, and percentages of breast, carcass, and heart of male broiler chickens at d 42 of age (n = 2 chickens per pen; 9 pens per treatment; LSMeans)

| Parameter | Incubation | | Macro minerals | | Trace minerals | | SEM | P values | | |
| --- | --- | --- | --- | --- | --- | --- | --- | --- | --- | --- |
|  | Darkness | Green | Inorganic | Organic | Inorganic | Organic |  | Incubation | Macro minerals | Trace minerals |
| Body weight^1^ (g) | 3,054^b^ | 3,130^a^ | 3,117 | 3,066 | 3,058 | 3,125 | 26 | 0.05 | 0.18 | 0.08 |
| Breast weight^2^ (%) | 21.8 | 21.7 | 21.8 | 21.8 | 21.7 | 21.8 | 0.7 | 0.39 | 0.50 | 0.21 |
| Carcass weight^2,3^ (%) | 53.2 | 52.8 | 53.1 | 53.0 | 53.3 | 52.8 | 1.3 | 0.30 | 0.26 | 0.52 |
| Heart weight^2^ (%) | 0.5 | 0.5 | 0.5 | 0.5 | 0.5 | 0.5 | 0.001 | 0.65 | 0.73 | 0.50 |

^1^ Average body weight of slaughtered chickens at d 42.

^2^ Percentages of pectoralis major plus pectoralis minor and hearth weight of average body weight.

^3^ Carcass weight (feathers and skin are attached) without both wings (detached from humerus head) and legs (detached from femur head).

^a-b^ Values within a row and factor lacking a common superscript differ (*P* ≤ 0.05).

Table A3. Effects of green LED light during incubation and dietary macro and trace mineral source during rearing on home pen behaviour of male broilers (eating, drinking, walking, standing, resting, foraging, comfort behaviour, dustbathing, and perching, expressed as percentage of chickens performing each behaviour) for each of the scanning days (day 6, 13, 20, 27, 34, and 41).

| Parameter and day | Incubation | | Macro minerals | | Trace minerals | | SEM | *P*-values | | |
| --- | --- | --- | --- | --- | --- | --- | --- | --- | --- | --- |
|  | Darkness | Green | Inorganic | Organic | Inorganic | Organic |  | Incubation | Macro minerals | Trace minerals |
| Eating |  |  |  |  |  |  |  |  |  |  |
| 6 d | 21.8 | 19.8 | 22.3 | 19.4 | 18.1^b^ | 23.5^a^ | 1.7 | 0.40 | 0.23 | 0.03 |
| 13 d | 19.8 | 22.5 | 21.2 | 21.1 | 22.3 | 19.9 | 1.2 | 0.11 | 0.95 | 0.17 |
| 20 d | 18.1 | 22.0 | 21.3 | 18.9 | 19.6 | 20.6 | 2.5 | 0.28 | 0.51 | 0.78 |
| 27 d | 19.9 | 18.5 | 17.6 | 20.8 | 20.3 | 18.1 | 2.5 | 0.71 | 0.39 | 0.54 |
| 34 d | 19.1 | 20.1 | 20.1 | 19.1 | 19.9 | 20.0 | 1.7 | 0.45 | 0.45 | 0.98 |
| 41 d | 18.7 | 23.4 | 22.1 | 20.0 | 20.3 | 21.8 | 1.9 | 0.09 | 0.43 | 0.57 |
| Drinking |  |  |  |  |  |  |  |  |  |  |
| 6 d | 12.2 | 10.7 | 11.8 | 11.2 | 11.0 | 12.0 | 1.3 | 0.42 | 0.77 | 0.59 |
| 13 d | 10.6 | 12.8 | 10.2 | 13.3 | 12.3 | 11.1 | 1.5 | 0.30 | 0.15 | 0.58 |
| 20 d | 13.1 | 16.1 | 12.7 | 16.5 | 15.2 | 13.9 | 2.1 | 0.33 | 0.22 | 0.68 |
| 27 d | 15.7 | 14.3 | 13.7 | 16.3 | 15.5 | 14.5 | 2.3 | 0.67 | 0.42 | 0.77 |
| 34 d | 12.9 | 13.4 | 15.8 | 12.7 | 12.1 | 14.3 | 1.6 | 0.84 | 0.08 | 0.32 |
| 41 d | 13.8 | 13.8 | 13.5 | 14.0 | 14.2 | 13.4 | 1.7 | 0.99 | 0.84 | 0.73 |
| Walking |  |  |  |  |  |  |  |  |  |  |
| 6 d | 16.3 | 11.4 | 14.1 | 13.7 | 13.9 | 13.8 | 1.7 | 0.06 | 0.87 | 0.96 |
| 13 d | 17.1 | 15.1 | 16.4 | 15.8 | 16.7 | 15.5 | 1.7 | 0.42 | 0.78 | 0.62 |
| 20 d | 20.7 | 17.0 | 20.3 | 17.3 | 18.2 | 19.5 | 2.1 | 0.22 | 0.31 | 0.67 |
| 27 d | 21.2 | 18.2 | 20.0 | 19.4 | 19.7 | 19.6 | 2.2 | 0.35 | 0.85 | 0.98 |
| 34 d | 15.5 | 14.5 | 15.8 | 14.2 | 15.9 | 14.1 | 1.8 | 0.72 | 0.55 | 0.49 |
| 41 d | 20.3 | 18.5 | 17.4 | 17.3 | 18.4 | 16.4 | 1.8 | 0.21 | 0.98 | 0.44 |
| Standing |  |  |  |  |  |  |  |  |  |  |
| 6 d | 16.4 | 18.7 | 17.7 | 17.4 | 17.7 | 17.4 | 1.9 | 0.41 | 0.93 | 0.93 |
| 13 d | 16.1 | 16.9 | 18.1 | 14.9 | 15.2 | 17.9 | 1.8 | 0.72 | 0.21 | 0.28 |
| 20 d | 18.4 | 20.1 | 19.8 | 18.7 | 22.3 | 16.2 | 2.2 | 0.59 | 0.73 | 0.06 |
| 27 d | 17.2 | 19.4 | 19.2 | 17.5 | 17.2 | 19.5 | 2.3 | 0.49 | 0.62 | 0.47 |
| 34 d | 15.5 | 14.6 | 14.1 | 16.0 | 14.8 | 15.4 | 1.9 | 0.74 | 0.50 | 0.83 |
| 41 d | 18.7 | 22.9 | 20.8 | 20.8 | 20.9 | 20.6 | 2.0 | 0.15 | 0.99 | 0.90 |
| Resting |  |  |  |  |  |  |  |  |  |  |
| 6 d | 34.3 | 31.0 | 30.6 | 34.8 | 35.7 | 29.7 | 2.6 | 0.36 | 0.25 | 0.10 |
| 13 d | 33.7 | 28.8 | 31.3 | 31.3 | 29.9 | 32.6 | 2.7 | 0.19 | 0.99 | 0.49 |
| 20 d | 24.5 | 22.3 | 23.2 | 23.5 | 21.0 | 25.7 | 2.2 | 0.49 | 0.94 | 0.15 |
| 27 d | 20.9 | 26.7 | 24.6 | 23.0 | 23.0 | 24.5 | 2.2 | 0.07 | 0.63 | 0.65 |
| 34 d | 33.5 | 32.8 | 30.3 | 35.9 | 33.5 | 32.8 | 2.7 | 0.88 | 0.14 | 0.86 |
| 41 d | 27.5 | 22.4 | 24.5 | 25.7 | 24.3 | 25.9 | 2.3 | 0.14 | 0.70 | 0.62 |
| Forage |  |  |  |  |  |  |  |  |  |  |
| 6 d | 0.9 | 0.7 | 0.9 | 0.7 | 1.2 | 0.5 | 0.04 | 0.69 | 0.72 | 0.24 |
| 13 d | 0.3 | 0.6 | 0.3 | 0.6 | 0.7 | 0.08 | 0.03 | 0.50 | 0.50 | 0.09 |
| 20 d | 3.7 | 0.9 | 1.4 | 3.3 | 2.8 | 1.9 | 0.09 | 0.05 | 0.17 | 0.52 |
| 27 d | 3.7 | 1.4 | 2.8 | 2.4 | 2.8 | 2.3 | 0.1 | 0.11 | 0.79 | 0.75 |
| 34 d | 0.7 | 1.7 | 0.9 | 1.5 | 1.0 | 1.5 | 0.06 | 0.25 | 0.47 | 0.54 |
| 41 d | 0.4 | 0.4 | 0.5 | 0.1 | 0.1 | 0.4 | 0.03 | 0.34 | 0.37 | 0.33 |
| Comfort behaviour |  |  |  |  |  |  |  |  |  |  |
| 6 d | 2.1 | 2.2 | 2.5 | 1.8 | 0.7 | 2.6 | 0.07 | 0.91 | 0.53 | 0.37 |
| 13 d | 2.7 | 3.3 | 2.8 | 3.2 | 2.8 | 3.2 | 0.08 | 0.65 | 0.77 | 0.77 |
| 20 d | 1.7 | 1.8 | 1.4 | 2.1 | 1.2 | 2.4 | 0.07 | 0.96 | 0.57 | 0.28 |
| 27 d | 1.6 | 1.6 | 2.3 | 0.8 | 1.59 | 1.54 | 0.07 | 0.97 | 0.17 | 0.97 |
| 34 d | 2.2 | 2.2 | 2.4 | 1.9 | 2.6 | 1.7 | 0.06 | 0.96 | 0.65 | 0.35 |
| 41 d | 1.2 | 2.6 | 1.5 | 2.3 | 2.2 | 1.7 | 0.07 | 0.18 | 0.40 | 0.66 |
| Dust bathing |  |  |  |  |  |  |  |  |  |  |
| 6 d | 0.9 | 0.7 | 0.5 | 1.2 | 0.9 | 0.7 | 0.05 | 0.77 | 0.29 | 0.80 |
| 13 d | 0 | 0.3 | 0 | 0.3 | 0.3 | 0.1 | 0.01 | 0.31 | 0.31 | 0.34 |
| 20 d | 0 | 0 | 0 | 0 | 0 | 0 | 0 | 0 | 0 | 0 |
| 27 d | 0 | 0 | 0 | 0 | 0 | 0 | 0 | 0 | 0 | 0 |
| 34 d | 1.0 | 0.2 | 0.1 | 0.9 | 0.5 | 0.5 | 0.03 | 0.11 | 0.10 | 0.99 |
| 41 d | 0 | 0 | 0 | 0 | 0 | 0 | 0 | 0 | 0 | 0 |

a-b Values within a row, lacking a common superscript differ (*P* ≤ 0.05).

Due to same scores in each treatment group for perching, values are not shown.
